# Extensive sucrose cycling through the fructan pool of a C_3_ grass across a 200 to 800 μmol mol^−1^ atmospheric CO_2_ gradient

**DOI:** 10.1093/plphys/kiag381

**Published:** 2026-06-16

**Authors:** Jianjun Zhu, Christoph A Lehmeier, Ulrike Ostler, Regina T Hirl, Juan C Baca Cabrera, Rudi Schäufele, Fernando A Lattanzi, Hans Schnyder

**Affiliations:** Technische Universität München, Lehrstuhl für Grünlandlehre, Alte Akademie 12, 85354 Freising-Weihenstephan, Germany; State Key Laboratory of Microbial Technology, Shandong University, Qingdao, Shandong, China; Technische Universität München, Lehrstuhl für Grünlandlehre, Alte Akademie 12, 85354 Freising-Weihenstephan, Germany; Technische Universität München, Lehrstuhl für Grünlandlehre, Alte Akademie 12, 85354 Freising-Weihenstephan, Germany; Karlsruhe Institute of Technology, Institute of Meteorology and Climate Research, Atmospheric Environmental Research (IMKIFU), Garmisch-Partenkirchen, Germany; Technische Universität München, Lehrstuhl für Grünlandlehre, Alte Akademie 12, 85354 Freising-Weihenstephan, Germany; Technische Universität München, Lehrstuhl für Grünlandlehre, Alte Akademie 12, 85354 Freising-Weihenstephan, Germany; Forschungszentrum Jülich GmbH, Institute of Bio- and Geoscience: Agrosphere (IBG-3), Jülich, Germany; Technische Universität München, Lehrstuhl für Grünlandlehre, Alte Akademie 12, 85354 Freising-Weihenstephan, Germany; Technische Universität München, Crop Physiology, Freising-Weihenstephan, Germany; Area de Pasturas y Forrajes, Instituto Nacional de Investigación Agropecuaria (INIA), La Estanzuela, Colonia, Uruguay; Grupo Disciplinario Produccion de Pasturas, Departamento de Produccion Animal, Facultad de Agronomia, Universidad de la Republica, Montevideo, Uruguay; Technische Universität München, Lehrstuhl für Grünlandlehre, Alte Akademie 12, 85354 Freising-Weihenstephan, Germany

## Abstract

Fructan stores constitute the most important reserve carbohydrate pools of cool season (C_3_) grasses, but it is largely unknown how the variation of atmospheric CO_2_ concentration ([CO_2_]) between past and projected future levels affects their function in source (photosynthesis)–sink (growth and respiration) relationships. This study addressed this question with perennial ryegrass (*Lolium perenne* L.) grown at [CO_2_] of 200, 400, or 800 μmol mol^−1^ with growth-limiting nitrogen fertilization. Sixty-six days-old vegetative stands were labelled with ^13^CO_2_/^12^CO_2_ mixtures for 7 days and the ^13^C-tracer dynamics in whole-shoot sucrose, fructans, glucose, and fructose pools evaluated with a 4-pool compartmental model of central carbohydrate metabolism. Increasing [CO_2_] from 200 to 800 μmol mol^−1^ increased shoot mass 1.6-fold and fructan concentration from 35% to 50% of dry weight, while decreasing the nitrogen nutrition status of stands. Conversely, [CO_2_] had no effect on the half-lives of water-soluble carbohydrate pools (fructans, ∼7.7 days; sucrose, glucose, and fructose, 2.3 to 4.5 h). Carbon cycling through the fructan pool was enhanced by [CO_2_], increasing the mean residence time of carbon in the carbohydrate system from 9 to 14 d between 200 and 800 μmol mol^−1^ [CO_2_]. Sucrose resynthesis from breakdown products of fructans (fructose) was extensive and corresponded to 65% and 113% of concurrent sucrose neo-synthesis (or sucrose consumption in growth and respiration) at 200 and 800 μmol mol^−1^ [CO_2_], respectively. These results imply a strong constitutive buffering of sucrose availability in the source–sink system of perennial ryegrass (and likely most other C_3_ grasses) by fructan metabolism.

## Introduction

Plants' physiological resilience—that is their ability to withstand and perform under stress (eg drought) or in response to disturbance (eg defoliation)—depends critically on the presence and function of nonstructural carbon (NSC) stores, most commonly in the form of starch, sugars, or fructans (eg Pollock and Cairns 1991; Martínez-Vilalta et al. 2016; Smith and Zeeman 2020; Signori-Müller et al. 2021). Such a role is connected to the plants' ability to adjust the partitioning of photosynthetic products to NSC stores, increasing it in times when photosynthesis is greater than demand by growth, maintenance, and defense processes, and depleting and using it when photosynthetic activity is insufficient (eg Volenec 1986; Galiano et al. 2011; and citations above).

Fructans are found in ca. 15% of plant species (Hendry 1993), mainly in the families *Poaceae* and *Liliaceae* (monocots; Van den Ende et al. 2004) and in the *Asteraceae*, *Campanulaceae*, and *Boraginaceae* (dicots) (Versluys et al. 2018). Specifically, in most cool season (C_3_) grasses, including wheat, barley, and many wild and cultivated forage grasses, NSC stores are predominantly composed of fructans (Nelson and Spollen 1987; Pollock and Cairns 1991; Versluys et al. 2018) and play key roles in defoliation tolerance (eg Meuriot et al. 2018) and grain filling (eg Schnyder 1993; Gebbing and Schnyder 1999). Fructans are water-soluble fructose polymers synthesized from sucrose (Wagner et al. 1983), stored in vacuoles (Wagner et al. 1983), turned over relatively slowly (half-life >1 d; Lattanzi et al. 2012), and can be found in all plant parts of fructan-storing grasses, including developing and mature leaf blades and leaf sheaths (Pollock and Cairns 1991). Consistent with the source–sink dynamics concept (eg Martínez-Vilalta et al. 2016), fructan stores are generally increased in conditions of nutrient limitation, high irradiance, mild drought, low temperature, or elevated atmospheric CO_2_ concentration ([CO_2_]) (Pollock and Cairns 1991; Hendry 1993; Smart et al. 1994; Barnes et al. 1995; Versluys et al. 2018). However, it is unknown if very low [CO_2_], such as experienced during the Last Glacial Maximum [CO_2_] ∼180 μmol mol^−1^ (Lüthi et al. 2008), may perhaps impair the C_3_ grasses' (or other species') ability to store (and utilize) fructans.

The function of NSC stores in receiving photosynthetic products and supplying substrate for growth and respiration of intact plants is best investigated by quantitative carbon (C) isotopic labelling of the photosynthetic assimilates and analysis of the tracer kinetics in the system by means of compartmental modeling (eg Lehmeier et al. 2008; Lattanzi et al. 2012; Ostler et al. 2016). Such investigations indicated that NSC stores are a constitutive part of central carbohydrate metabolism, in the sense that they turn over continuously (Ostler et al. 2016), even in nondisturbed conditions with continuous photosynthetic activity (Lattanzi et al. 2012). In perennial ryegrass (*Lolium perenne* L.) this NSC store is situated in the shoot, predominantly composed of fructans, and supplied >50% of the total amount of substrate for both shoot and root respiration (Lehmeier et al. 2008) and tissue production in leaf growth zones of both dominant and subordinate individuals in plant stands (Lattanzi et al. 2005).

**Figure 1:**
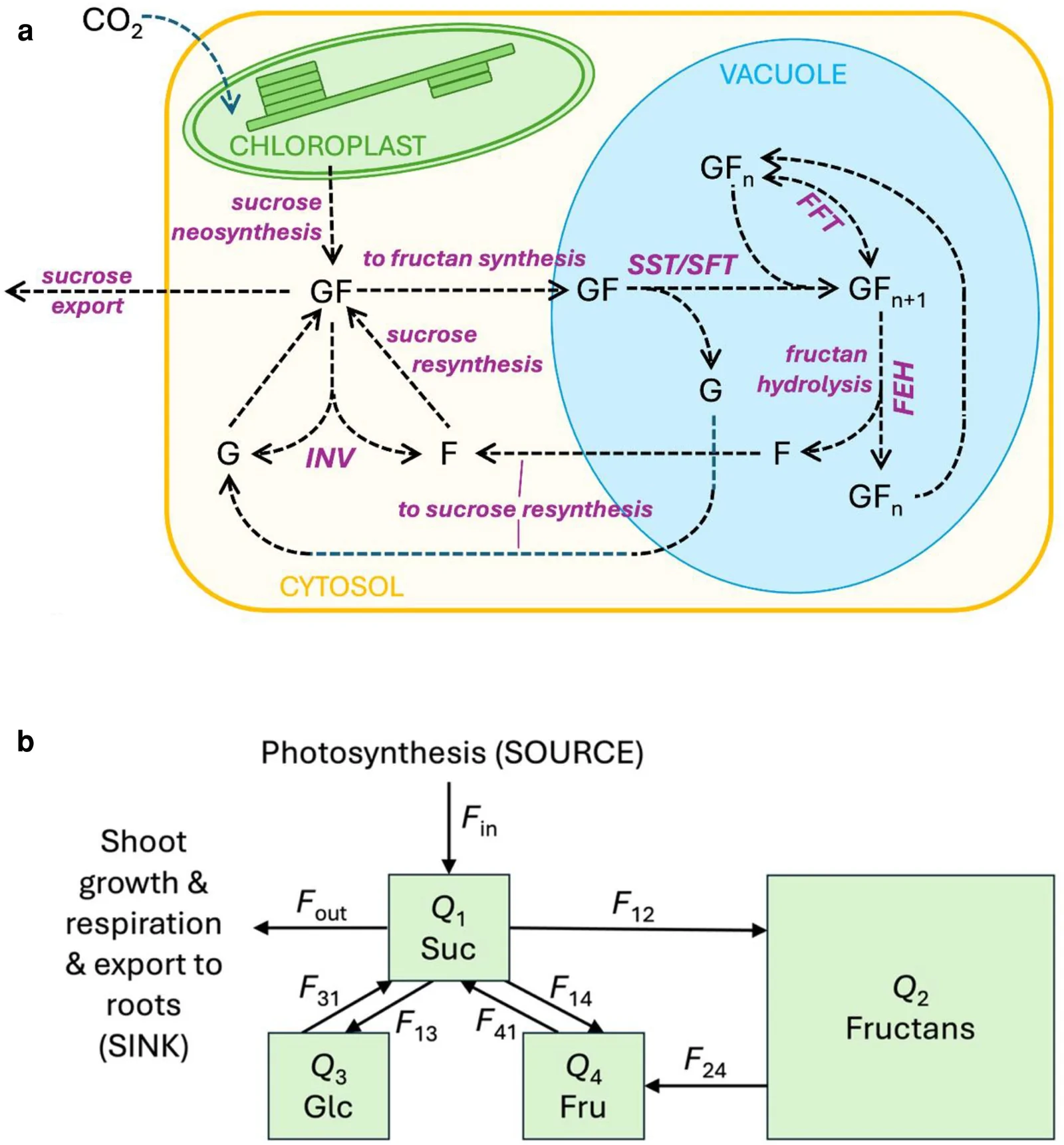
(a) Simplified scheme of central carbohydrate metabolism of a photosynthetically active cell in a fructan-storing plant, highlighting sucrose consumption and resynthesis associated with futile cycling (involving invertase [INV] activity) and fructan metabolism. G and F represent free glucose and fructose, or the glucosyl and fructosyl moieties of sucrose (GF) or fructan molecules (GF_n_), with *n* the number of fructosyl units in a molecule. Fructan synthesis occurs by the coordinated activities of sucrose:sucrose fructosyl transferase(s) (SST, which catalyzes the transfer of the fructosyl residue of a donor sucrose molecule onto an acceptor sucrose molecule, generating a fructan trisaccharide (GFF) and liberating G: GF + GF → G + GFF) and sucrose:fructan fructosyl transferase (SFT, which catalyzes the transfer of the fructosyl residue of a donor sucrose molecule onto an acceptor fructan molecule: GF + GF_n_ → G + GF_n+1_). Fructan:fructan fructosyl transferase(s) (FFT) catalyze(s) the transfer of individual fructosyl residues between fructan molecules. Fructosyl units are cleaved from (terminal ends) of fructan molecules by fructan:fructose exohydrolase (FEH) activity, thus liberating fructose for sucrose resynthesis. Invertase activity may also be present in the vacuole (and apoplast) but is not highlighted here. Several metabolic processes—particularly sucrose neo-synthesis (de novo synthesis) and sucrose resynthesis from glucose and fructose—are multienzymatic processes, not specified in detail in the figure. (b) Four-pool compartmental model of central carbohydrate metabolism in a fructan storing plant (adapted from Lattanzi et al. 2012). Suc, sucrose; Glc, glucose; Fru, fructose. *Q*_i_ represents the size of a carbohydrate pool *i*, and *F*_ij_ is the flux from the donor pool *i* to the receiving pool *j*. Thus, *F*_12_ denotes the fructosyl transferase–catalyzed transfer of the fructosyl residue of sucrose to an acceptor (sucrose or fructan) molecule in the fructan pool; *F*_13_, glucose production by fructosyl transferases or invertase(-like) activities; *F*_14_, fructose production by invertase(-like) activity; *F*_24_, cleavage of fructose from fructans by fructan exohydrolase activity; *F*_31_, glucose use in sucrose resynthesis; and *F*_41_ fructose use in sucrose resynthesis. *F*_in_ denotes the flux of photosynthetically neo-synthesized sucrose into the system, and *F*_out_, the efflux of sucrose from the system for consumption in shoot growth and respiration plus export to roots. For mathematical details of the model, including other functional model parameters (eg pool half-life or residence time of C in the carbohydrate system), see Theory and model, Materials and methods, and Table S1. As we show below, the above 4-pool model “constrained” with a 1:1 stoichiometric ratio of glucose and fructose use in resynthesis of sucrose and without glucose–fructose interconversions (see Table S1) provided the most parsimonious best fit to our experimental results.

Sucrose plays a unique role in central metabolism of C_3_ grasses, as in most plants (Fig. 1a): it is (i) the main carbohydrate synthesized in the cytoplasm of photosynthetic tissue (Sharkey 2024), (ii) an important diurnal storage carbohydrate in vacuoles (Kaiser et al. 1982; Farrar and Farrar 1986; Gerhardt et al. 1987), and (iii) the predominant form of carbohydrate translocation to sink and storage tissues (Lalonde et al. 2004; Braun 2022). Moreover, (iv) hydrolysis by invertase(s) (possibly including minor invertase-like activity, eg from fructosyl transferase(s) or fructan exohydrolase(s); eg Sprenger et al. 1995; Nagaraj et al. 2004; Le Roy et al. 2008) is a potentially very strong sink for sucrose in both sink and source tissue (Huber 1989; Geigenberger and Stitt 1991; Kingston-Smith et al. 1999; Nguyen-Quoc and Foyer 2001). Most importantly, in most C_3_ grasses and other fructan storing species, (v) sucrose is the immediate substrate for fructan synthesis and (vi) the form into which fructan breakdown products are regenerated (Wagner et al. 1986; Wagner and Wiemken 1987; Pollock and Cairns 1991). Clearly, understanding of fructan dynamics requires a consideration of its integration in sucrose metabolism, including all of the above relationships (see also Theory and model and Fig. 1). However, present knowledge about these relationships is very limited, particularly at the scale of whole plants. Furthermore, how this integration is achieved at very low [CO_2_] or predicted future [CO_2_] is entirely unknown.

To shed light on these knowledge gaps, we experimentally determined the effects of [CO_2_] during growth on (i) the concentrations of sucrose, fructans, glucose, and fructose in whole shoots of perennial ryegrass, and estimated (ii) the half-life of these carbohydrate pools, (iii) the partitioning of sucrose to fructan synthesis vs growth + respiration (including export to roots) vs hydrolysis by invertase-like activities, (iv) the de novo synthesis of sucrose vs its recycling via the fructan pool, and (v) the residence time of C in the whole-shoot carbohydrate system. This was achieved by mesocosm-scale dynamic ^13^CO_2_/^12^CO_2_-labelling experiments (Schnyder et al. 2003) of well-developed 66-days-old stands of *L. perenne* grown at 200, 400, or 800 μmol mol^−1^ [CO_2_] at 20/16 °C during the 16/8-h day/night periods and a photosynthetic photon flux density (PPFD) of 800 µmol m^−2^ s^−1^ at canopy height during the light period. We employed compartmental modeling (Fig. 1) to the tracer kinetics in sucrose, fructans, glucose, and fructose, which were extracted from whole shoots of perennial ryegrass sampled at the end of light periods at intervals ranging from one photoperiod to 7 d during dynamic labelling. In this we also tested 4-pool model variants with different biochemical complexity (Figure S1), as in Lattanzi et al. (2012).

We grew the stands with a nitrogen (N)-reduced Hoagland-type nutrient solution, known to cause N-limited growth (eg Lehmeier et al. 2013; Baca Cabrera et al. 2020), since most grasslands display N-limited growth even with relatively high rates of N fertilizer addition (eg Lüscher et al. 2004; Baca Cabrera et al. 2021). Therefore, as an ancillary objective, we also determined the effect of [CO_2_] on N nutritional status of the stands (Lemaire et al. 2008), which is known to respond to [CO_2_] (Lüscher et al. 2004; Baca Cabrera et al. 2021; Bassiouni et al. 2025) and might interactively affect fructan metabolism.

## Theory and model

Here we use a 4-pool compartmental model of central carbohydrate metabolism, and its integration in the whole plant source–sink system, to explore the specific role of fructan metabolism in sucrose consumption and resynthesis (cycling) and related metabolism in perennial ryegrass at different [CO_2_] levels (Fig. 1). This model was originally devised for the mature leaf blade of perennial ryegrass (Lattanzi et al. 2012) and is here applied to the entire vegetative shoot (Fig. 1b). The latter is composed of vegetative tillers, which themselves are made up of a series of leaves—including their blade and sheath parts—across the full developmental range from immature expanding to mature and senescent (eg Schnyder and de Visser 1999). Hence, the model is effectively used as a whole-leaf model that integrates across all developmental stages of leaves comprised in the vegetative shoot.

The model (including its variants, Table S1) is based on common assumptions of compartmental models (eg Lattanzi et al. 2005), namely that (i) the system is in a steady state at the day-by-day timescale, (ii) fluxes obey first-order kinetics, and (iii) pools are homogeneous and well mixed, so that the flux(es) out of and into a pool, as well as the pool's size and half-life, are constant. For an evaluation of the assumptions and simplifications of the model, see Discussion.

Based on knowledge of carbohydrate metabolism in C_3_ grasses (Lattanzi et al. 2012; see also Fig. 1a), the model (Fig. 1b) accounts for the facts that:

1. current photosynthesis supplies the system via the sucrose pool (*F*_in_),
2. sucrose is consumed in 3 types of processes:
  a. growth and respiration, including export to roots (*F*_out_; with the magnitude of *F*_out_ equivalent to *F*_in_ in the steady state),
  b. fructan synthesis (with the fructosyl moiety of a donor sucrose molecule transferred to an acceptor sucrose or fructan molecule [*F*_12_]), with concurrent release of one glucose molecule (generating a portion of the flux *F*_13_, equivalent to *F*_12_),
  c. sucrose hydrolysis by invertase(-like) processes generating equimolar amounts of fructose (*F*_14_) and glucose (with glucose production in this process equivalent to *F*_13_–*F*_12_),
3. fructan hydrolysis produces fructose (*F*_24_, equivalent to *F*_12_ in the steady state), and
4. fructose (*F*_41_) and glucose (*F*_31_) are (re)utilized in sucrose resynthesis, with a 1:1 stoichiometry of fructose and glucose use in this process. Therefore, *F*_41_ = *F*_31_.

To reduce complexity and to enhance statistical robustness of the model, we ignore the use of sucrose as starter molecule in fructan synthesis and the production of sucrose or glucose during fructan hydrolysis as these are minor fluxes in species with a high degree of polymerization (DP) of fructans such as *L. perenne* (Pavis et al. 2001). Consideration of sucrose use as a starter molecule (acceptor sucrose molecule in the sucrose:sucrose fructosyl transferase (SST) reaction; Fig. 1a), would increase slightly the actual stoichiometry of the overall fluxes associated with fructan synthesis (hexose units added to the fructan pool per unit glucose released in the fructosyl transferase reactions is slightly greater than 1:1). On balance, however, in a steady state, with rates of fructan synthesis equal to fructan degradation, use of sucrose as a starter molecule for fructan synthesis is compensated by release.

Predictions by the model include (i) the half-life of the pools of sucrose, fructans, glucose, and fructose, (ii) the fluxes of C through each of these pools, (iii) sucrose partitioning between fructan synthesis, hydrolysis, and use in growth and respiration (including export to roots), and (iv) the residence time of C in the whole-shoot carbohydrate system. These predictions are optimized to fit the tracer kinetics in the different carbohydrate pools best, while the observed whole-shoot concentrations of sucrose, fructans, glucose, and fructose constrain the pool sizes (analogous to Lattanzi et al. 2012). A role of (diurnal) starch stores is not specified in the model—as *L. perenne* leaves are known to contain only trace amounts of starch even when induced to accumulate carbohydrates (Turner et al. 2002; see also Wagner et al. 1983)—but is principally implicit in the (nocturnal) substrate supply to the system via sucrose neo-synthesis (Sharkey 2024).

Delays in the system caused by vascular or extravascular transport between and within organs composing the shoot are neglected as these are expected to be very short (<1 h), particularly in comparison to the kinetics of the fructan pool. For instance, Lehmeier et al. (2008), working with the same cultivar under similar conditions, observed a 0.8-h transport delay between shoot and root for transport of respiratory substrate. Such a delay is consistent with current understanding of phloem transport velocity (Lalonde et al. 2004; Jensen et al. 2012; Braun 2022). In contrast, the fructan pool of leaf blades had a half-life of >1 d (Lattanzi et al. 2012).

Despite being an abstraction, the model displayed in Fig. 1 is biologically realistic in important ways (compare Fig. 1a and b). Firstly, it places the shoot sucrose pool in the center of the carbohydrate system, which is fed by photosynthesis and supplies growth and respiration in the shoot and export to the roots. Particularly, in grasses (beyond roots) the growing and differentiating shoot tissues are completely heterotrophic (eg Schnyder and Nelson 1987), thus fully dependent on the supply of sucrose via the phloem. Secondly, essentially the entire carbohydrate system supplying growth and respiration is situated in the shoot (Lehmeier et al. 2008). Thirdly, the model is mechanistically explicit in representing separately the major alternative sinks for sucrose within the carbohydrate system, namely the activities connected with cycling through either the fructan pool or through sucrose hydrolysis/resynthesis (futile cycling).

## Results

### No chamber effects

We observed no chamber effect on biomass, leaf area, and carbohydrate contents, since there was no statistically significant difference in any of these parameters for plants that were grown in different growth chambers operated at the same environmental settings (Table S2). Also, in the same experiments, Baca Cabrera et al. (2020) observed no chamber effect on any of a large suite of leaf morphophysiological parameters (including net photosynthesis, stomatal conductance, and leaf and epidermal cell dimensions).

### Plant biomass, leaf area, and canopy N status

Elevating [CO_2_] from 200 to 400 or 800 μmol mol^−1^ increased the C mass of whole plants by 34% or 59% (both *P* < 0.001) (Table 1). Meanwhile the shoot:root ratio was not affected significantly by [CO_2_] (Table 1). Leaf area per plant did not differ significantly between 200 and 400 μmol mol^−1^ [CO_2_], but decreased by 14% at 800 μmol mol^−1^ [CO_2_] relative to 200 μmol mol^−1^ [CO_2_] (*P* < 0.05). The N nutrition index (NNI) indicated N-limited growth in all treatments, with limitation strongly aggravated by increasing [CO_2_] (Table 1). Thus, the NNI of the plant canopies averaged 0.79 at 200 μmol mol^−1^ CO_2_ and decreased to 0.49 at 400 μmol mol^−1^ CO_2_ and 0.38 at 800 μmol mol^−1^ CO_2_ (both *P* < 0.001). Accounting for treatment-dependent dilution of N by the presence of water-soluble carbohydrates (WSC) by using a treatment-independent WSC concentration of 20% (w/w) still indicated a growth-limiting NNI_corr_ in all treatments, with NNI_corr_ still significantly decreasing with [CO_2_].

**Table 1:** Plant, shoot and root mass, shoot:root ratio, N nutrition index (NNI), and leaf area of perennial ryegrass (*Lolium perenne* L.) grown at 200, 400, or 800 μmol mol^−1^ [CO_2_]. Means (±SE).

	Atmospheric CO_2_ concentration (μmol mol^−1^)		
	200	400	800
Mass			
Plant (g C plant^−1^)	1.61^a^ (0.06)	2.15^b^ (0.06)	2.57^c^ (0.06)
Shoot (g C plant^−1^)	1.37^a^ (0.05)	1.84^b^ (0.06)	2.17^c^ (0.06)
Root (g C plant^−1^)	0.24^a^ (0.02)	0.31^b^ (0.01)	0.40^c^ (0.01)
Shoot:root (g C g^−1^ C) ratio	6.17^a^ (0.44)	5.94^a^ (0.33)	5.44^a^ (0.17)
NNI	0.79^a^ (0.02)	0.49^b^ (0.03)	0.38^c^ (0.01)
NNI_corr_	0.90^a^ (0.03)	0.61^b^ (0.02)	0.51^c^ (0.02)
Leaf area (m^2^ plant^−1^)	0.035^a^ (0.001)	0.038^a^ (0.001)	0.030^b^ (0.001)

Biomass and leaf area data are based on a total of 24 (200 and 800 μmol mol^−1^ [CO_2_]) or 48 (400 μmol mol^−1^ [CO_2_]) replicates sampled during the labelling experiments. NNI determinations are based on 16 (200 and 800 μmol mol^−1^ [CO_2_]) or 32 (400 μmol mol^−1^ [CO_2_]) plants sampled on d 63, 3 days prior to the beginning of labeling. NNI_corr_ corrects the NNI for the “dilution” effect of the high and [CO_2_]-dependent WSC concentration (see Materials and methods). Different superscript letters within a row indicate statistically significant differences among CO_2_ treatments (Table S3). For details, see Materials and methods.

### Water-soluble carbohydrates

WSC concentrations (mg WSC-C g^−1^ biomass-C) in whole-shoot biomass were generally very high and increased with [CO_2_]. That is, WSC concentration in the shoot was 42% at 200 μmol mol^−1^ and 57% at 800 μmol mol^−1^. Elevating [CO_2_] from 200 to 400 or 800 μmol mol^−1^ increased the total mass of WSC in the shoot by 65% or 116% (both at *P* < 0.001) in association with increases of WSC concentration in shoot biomass of 23% or 37% (*P* < 0.001) (Table 2). Since leaf area per plant decreased somewhat with increasing [CO_2_] (Table 1), these relationships dictated that WSC contents per unit whole-shoot leaf area still increased by 66% or 147% when [CO_2_] was elevated from 200 to 400 or 800 μmol mol^−1^.

**Table 2:** Water-soluble carbohydrate mass and concentration in shoot biomass of perennial ryegrass (*Lolium perenne* L.) grown at 200, 400, or 800 μmol mol^−1^ [CO_2_]. Means (±SE).

	Atmospheric CO_2_ concentration (μmol mol^−1^)		
	200	400	800
Water-soluble carbohydrates			
Mass (g C plant^−1^)	0.573^a^ (0.036)	0.947^b^ (0.043)	1.240^c^ (0.065)
Concentration (mg C g^−1^ biomass C)	418^a^ (20)	515^b^ (11)	571^c^ (27)

Data are based on a total of 24 (200 and 800 μmol mol^−1^ [CO_2_]) or 48 (400 μmol mol^−1^ [CO_2_]) replicates sampled during the labelling experiments. Different superscript letters within a row indicate statistically significant differences among CO_2_ treatments (Table S3). For details, see Materials and methods.

**Figure 2:**
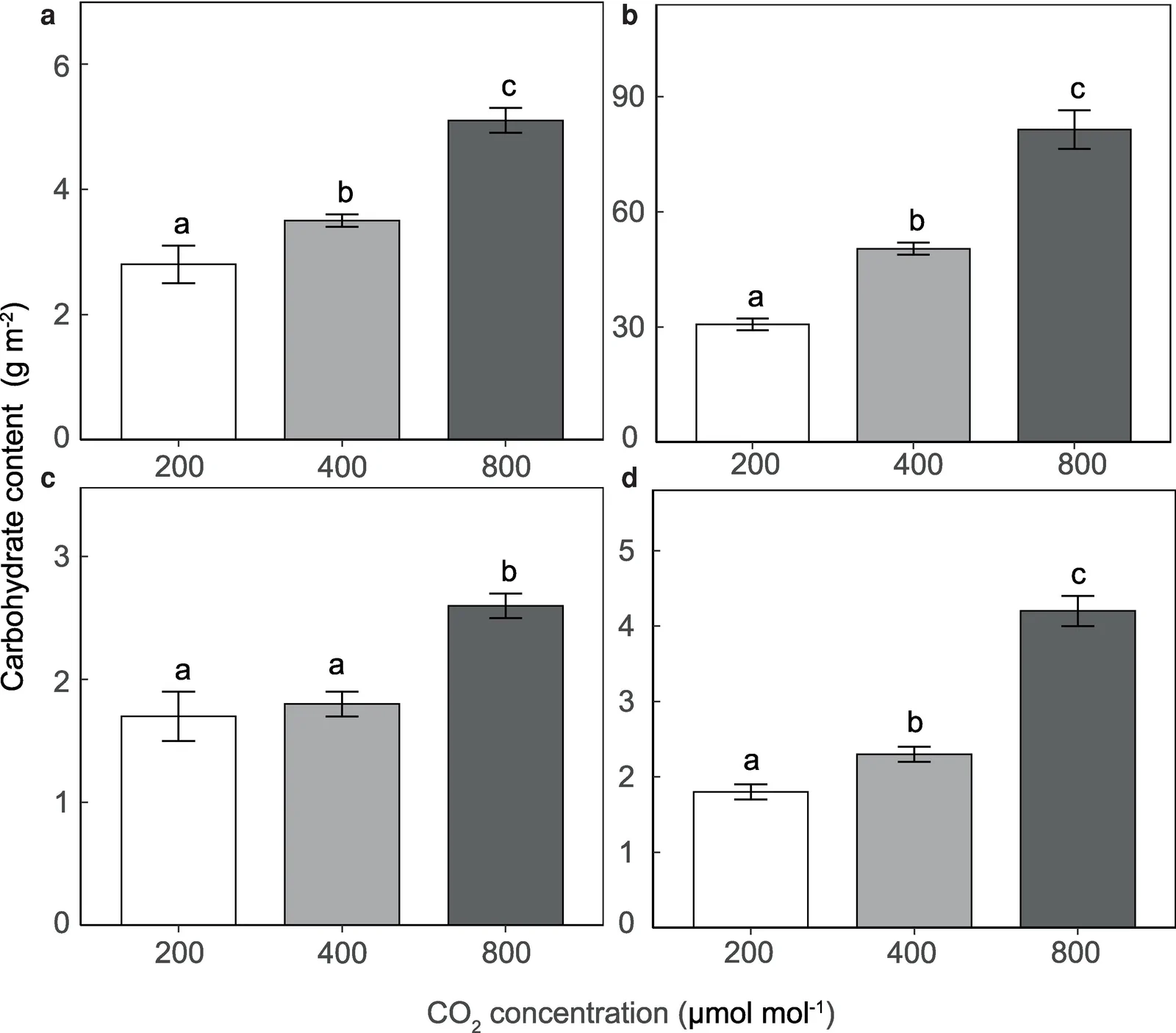
Contents of water-soluble carbohydrate-C per unit leaf area: sucrose (a), fructan (b), glucose (c), and fructose (d) of perennial ryegrass (*Lolium perenne* L.) plants as affected by atmospheric CO_2_ concentration during growth. The data are based on a total of 24 (200 and 800 μmol mol^−1^ CO_2_) or 48 (400 μmol mol^−1^ CO_2_) replicates sampled during the labelling experiments. Bars and error bars represent the means ± SE. Different superscript letters within a panel indicate statistically significant differences among [CO_2_] treatments at *P* < 0.001 (Table S3). For details, see Materials and methods.

Of all WSC fractions, fructan contents showed the strongest response to [CO_2_] (Fig. 2). Fructans accounted for 83–87% of total WSC in the different treatments and increased by 64% or 165% on a leaf area basis when [CO_2_] was elevated from 200 to 400 or 800 μmol mol^−1^ (Fig. 2). A [CO_2_] response of fructose was also observed, but was somewhat smaller, with fructose contents increasing by 28% or 133% when [CO_2_] was increased from 200 to 400 or 800 μmol mol^−1^. Sucrose and glucose contents also increased with [CO_2_], but those increases were distinctly smaller. The above relationships were stable during the 7-d-long labelling period (*P* > 0.10; Table S6).

### Compartmental analysis

**Figure 3:**
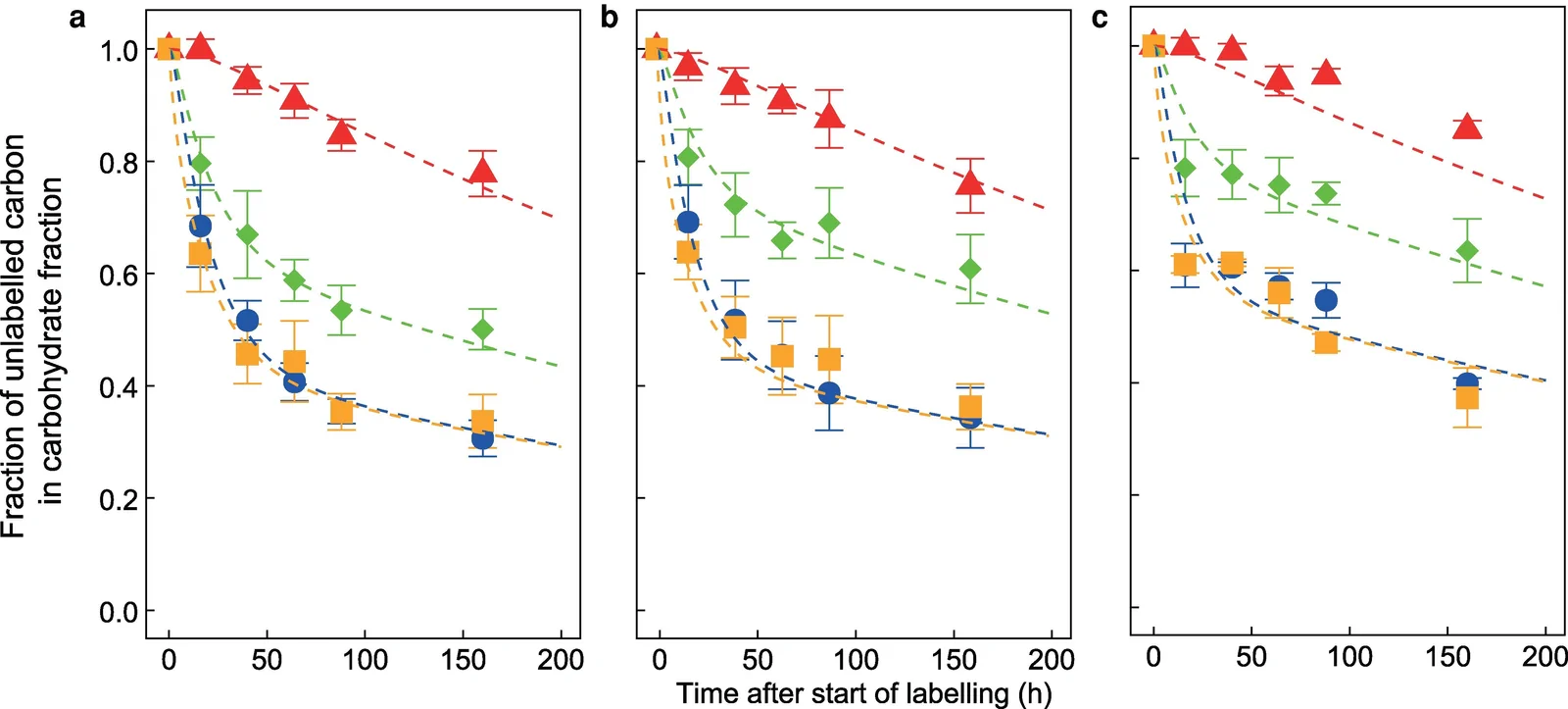
Time course of the fraction of unlabelled C (ƒ_unlab_) in fructans (red triangles), fructose (green diamonds), glucose (blue circles), and sucrose (orange squares) with labelling duration. Carbohydrates were extracted from the whole shoot of perennial ryegrass (*Lolium perenne*) grown at a [CO_2_] of 200 (a), 400 (b), or 800 μmol mol^−1^ (c). The curves represent the fits of the so-called constrained 4-pool compartmental model shown in Table S1. Each value is the mean (±SE) of 4 (200 and 800 μmol mol^−1^ [CO_2_]) or 8 (400 μmol mol^−1^ [CO_2_]) replicates.

The labelling time courses of the different carbohydrates (ie the decreases with time of the fraction of unlabelled *C* (*f*_unlab_) in a carbohydrate pool) were qualitatively similar in the different [CO_2_] treatments (Fig. 3): initial label incorporation—reflected in the initial decrease of *f*_unlab_ = (1—*f*_lab_)—was fastest and similar in sucrose and glucose, and slow in fructans. In comparison, fructose demonstrated a labelling time course that was intermediate between that of fructan and sucrose. In all [CO_2_] treatments, the time courses of *f*_unlab_ in the carbohydrate system of the whole shoot were best fitted with the so-called “stoichiometrically constrained” 4-pool compartmental model (Table 3 and Table S5). That is, by applying the Occam's razor principle, we identified as the “best” model the most parsimonious (ie simplest) model version (Fig. 1b, Table S1), which represented the data without a significant loss of statistical information based on the Bayesian Information Criterion (BIC; see Table S5). Thus, the additional parameters in the other model variants (3 out of 6 parameters in the “free model,” and 1 out of 6 parameters in the “without stoichiometric constraint model”; Table S5) were unnecessary in the sense that they did not improve statistically the model fit to the data. Accordingly, these other model variants “overfitted” the data; they were “overparameterized.” Conversely, the fitting parameters (n = 3) in the “constrained” model were all significant, identifying the “constrained” 4-pool model as the minimum model necessary for capturing all statistically essential information in the tracer data. This model dictated a 1:1 stoichiometry of glucose and fructose use in resynthesis of sucrose and did not consider glucose-fructose interconverting reactions (Fig. 1b, Table S1). The goodness of fit was near perfect for the 200 μmol mol^−1^ [CO_2_] treatment (*R*^2^ = 0.99) and 400 μmol mol^−1^ [CO_2_] treatment (*R*^2^ = 0.98), but somewhat less so for the 800 μmol mol^−1^ [CO_2_] treatment (*R*^2^ = 0.89).

**Table 3:** Functional characteristics of central carbohydrate metabolism (Fig. 1) in the shoot of perennial ryegrass (*Lolium perenne*) as affected by atmospheric CO_2_ concentration during growth: half-lives of carbohydrate pools (sucrose, fructans, glucose, fructose), C flux into (*F*_in_) and out (*F*_out_) of the carbohydrate system (via sucrose) and among carbohydrate pools (*F*_12_, sucrose to fructans; *F*_13_, sucrose to glucose; *F*_14_, sucrose to fructose; *F*_24_, fructans to fructose; *F*_31_, glucose to sucrose; *F*_41_, fructose to sucrose); sucrose partitioning between growth + respiration (including export to roots), fructan synthesis and hydrolysis (futile cycling); sucrose de novo and resynthesis fluxes; cycling, the fraction of sucrose cycling through fructans and sucrose hydrolysis–resynthesis (futile cycling); and mean residence time of C in the carbohydrate system.

	Atmospheric CO_2_ concentration (μmol mol^−1^)		
	200	400	800
	Pool half-life (T_1/2 Q1_, h)		
Sucrose	2.4^a^ (0.2)	2.5^a^ (0.2)	2.3^a^ (0.4)
Fructans	191^a^ (15)	182^a^ (16)	179^a^ (39)
Glucose	3.7^a^ (0.3)	3.3^a^ (0.3)	2.9^a^ (0.5)
Fructose	3.8^a^ (0.3)	4.3^a^ (0.3)	4.5^a^ (0.8)
	C flux (g m^−2^ h^−1^)		
*F*_in_ = *F*_out_	**0.17^a^ (0.02)**	**0.23^b^ (0.01)**	**0.29^b^ (0.04)**
*F*_12_ = *F*_24_	**0.11^a^ (0.01)**	**0.19^b^ (0.02)**	**0.32^b^ (0.07)**
*F*_13_	0.33^a^ (0.04)	0.38^a^ (0.03)	0.64^b^ (0.12)
*F*_14_	**0.22^a^ (0.03)**	**0.18^a^ (0.02)**	**0.32^a^ (0.10)**
*F*_31_ = *F*_41_ (= *F*_12_ + *F*_14_)	0.33^a^ (0.04)	0.38^a^ (0.03)	0.64^b^ (0.12)
	Sucrose partitioning (fraction)		
to growth and respiration	0.35^a,A^ (0.05)	0.38^a,A^ (0.03)	0.31^a,A^ (0.06)
to fructan synthesis	0.22^a,B^ (0.03)	0.32^b,A^ (0.04)	0.35^a,b,A^ (0.09)
to hydrolysis	0.43^a,A^ (0.07)	0.31^a,A^ (0.04)	0.34^a,A^ (0.12)
	Sucrose synthesis		
De novo (g m^−2^ h^−1^)*	0.17	0.23	0.29
Resynthesis (g m^−2^ h^−1^)^†^	0.66	0.74	1.30
Cycling (fraction)^‡^	0.80	0.76	0.82
	Residence time in the system (h)		
	213^a^ (24)	256^a,b^ (18)	323^b^ (46)
	Goodness of model fit (*R*^2^)		
	0.99	0.98	0.89

Parameters were estimated by fitting the constrained 4-pool compartmental model (compare with Table S1) to the tracer kinetics data shown in Fig. 3. Fluxes are expressed as g C m^−2^ leaf area h^−1^ and calculated with the equations given in Materials and methods by using the carbohydrate contents per leaf area as shown in Fig. 2. The results of the fluxes predicted by the optimized rate constants are given in bold. Small superscript letters indicate significant differences between CO_2_ treatments within a row (2-sample *z*-test, *P* < 0.05; exact pairwise *P* values are provided in Table S5-2). Capital letters (A, B) indicate approximate significant differences among the 3 sucrose partitioning fractions within the same CO_2_ column (2-sample *z*-test assuming independence; *P* < 0.05). Because the 3 fractions sum to 1, they are correlated; therefore, these comparisons are approximate and exploratory (see Table S5-1 for exact *P* values).

^*^Corresponds to *F*_in_.

^†^Sucrose resynthesis from products of sucrose hydrolysis and fructan degradation (*F*_31_ + *F*_41_).

^‡^The proportion of sucrose resynthesis (through fructan metabolism + futile cycling) in relation to total sucrose synthesis (ie neo-synthesis + resynthesis): (*F*_31_ + *F*_41_)/(*F*_in_ + *F*_31_ + *F*_41_).

[CO_2_] had no significant effect on the half-life of any of the carbohydrate pools (sucrose, fructans, glucose, or fructose) (Table 3). Also, at all [CO_2_] levels, the predicted half-lives were relatively short and similar for the sucrose (∼2.4 h), glucose (∼3 h), and fructose (∼4 h) pools, while that of the fructan pool (∼180 to 190 h) was almost 2 orders of magnitude higher than that of the sucrose pool (Table 3).

For the different [CO_2_] treatments, the model results indicated that about equal fractions of sucrose were partitioned between the 3 alternative functions: (1) supply of growth and respiration in the shoot *plus* export to roots or use in (2) fructan synthesis or (3) hydrolysis (Table 3). The approximate equal partitioning was true, except for the 200 μmol mol^−1^ [CO_2_] treatment, where sucrose partitioning to fructan synthesis (0.22) was lower than its use in the other processes (Table 3; Table S5-1). Also, partitioning to fructan synthesis at 200 μmol mol^−1^ [CO_2_] was lower than at 400 μmol mol^−1^ [CO_2_], although it did not differ significantly between 200 μmol mol^−1^ [CO_2_] and 800 μmol mol^−1^ [CO_2_] (Table 3; Table S5-2).

Particularly at 200 μmol mol^−1^ [CO_2_], sucrose use in hydrolysis was almost double that for fructan synthesis, while similar fractions were used in fructan synthesis and hydrolysis at 400 and 800 μmol mol^−1^ [CO_2_]. For a system in steady state, such a [CO_2_] treatment effect implies that sucrose recycling from the products of hydrolysis (futile cycling) was almost twice as important as that from fructose released during fructan breakdown at 200 μmol mol^−1^ [CO_2_], while at 400 and 800 μmol mol^−1^ [CO_2_], fructan metabolism and sucrose hydrolysis contributed approximately equally to sucrose recycling (Table 3). Overall, however, cycling (that is, the fraction of total sucrose synthesis that was connected to fructan metabolism + sucrose futile cycling [hydrolysis/resynthesis]) was remarkably constant among treatments at approximately 80% (Table 3).

Nevertheless, the treatments caused large differences in the magnitude of C fluxes within the system and out of the system. For instance, the total flux of sucrose out of the system (*F*_out_)—supplying shoot growth and respiration and export to roots—increased 1.7-fold between 200 and 800 μmol mol^−1^ [CO_2_]. The strongest treatment effect on C fluxes was connected to fructan synthesis (*F*_12_) and fructan hydrolysis (*F*_24_): increasing [CO_2_] from 200 to 800 μmol mol^−1^ increased the flux of C through the fructan pool 2.8-fold. On the other hand, we found no significant effect of [CO_2_] on the flux of sucrose through futile cycling (hydrolysis/resynthesis).

Meanwhile, the residence time of carbohydrate-C in the system increased from about 9 d to 14 d between 200 and 800 μmol mol^−1^ [CO_2_] in connection with greater sucrose partitioning to fructan synthesis (Table 3).

## Discussion

### Model realism

The 4-pool model of central carbohydrate metabolism depicted in Fig. 1 and its more complex variants (see Table S1) provided an almost perfect fit to the dynamic labelling data at 200 μmol mol^−1^ and 400 μmol mol^−1^ [CO_2_]. Such a good fit may not be intuitive given the abstractions and assumptions of the model (see Theory and model). However, in support of modelling assumptions, carbohydrate concentrations did not change during the 7-d-long dynamic labelling experiment (Table S6). Also, the shoot growth rate was <6% d^−1^ (Table S7), which implied that the actual rate of fructan synthesis (*F*_12_) on a given day overestimated the rate of hydrolysis (*F*_24_) by <6% d^−1^. In comparison, however, errors in model estimates for the other pools (sucrose, glucose, fructose) were much smaller, as they were turned over much faster. Also, the near-perfect fit of the model to the tracer data at 200 μmol mol^−1^ and 400 μmol mol^−1^ [CO_2_] implies that the biochemical (functional) features of central carbohydrate metabolism represented in the model must have been much more important than eventual effects of carbohydrate-specific compartmentation (at tissue, organ, or whole-shoot level), which were not considered in the model.

In that, the so-called “stoichiometrically constrained” model provided the most parsimonious best-fit to the labelling kinetics of the whole-shoot carbohydrate system at all [CO_2_] levels (Tables S1 and S3). In other words, the added biochemical complexity in the other tested model variants was statistically redundant for explaining the whole system labelling kinetics. That is, the effects of metabolic aspects not considered by the model were likely minor in comparison with the dominant features of the constrained 4-pool model (Table 3; Tables S1 and S3).

Although we know of no other studies of the half-lives of sucrose, glucose, or fructose at whole-shoot level (in *L. perenne*, or other *Poacea* species [such as wheat] or other plants), the estimates of those half-lives seem realistic when compared with studies at the scale of mature leaves (Farrar and Farrar 1986; Borland and Farrar 1988; Lattanzi et al. 2012) or leaf growth zones (Schnyder and Nelson 1987), in agreement with rapid sucrose transport times (Braun 2022) among leaf blades, sheaths, and immature growing tissue. Certainly, the idea that the sucrose pool is homogeneous is a gross simplification, as in grasses sucrose is generally present in more than one cellular compartment (cytoplasm and vacuole) in many tissues (eg Wagner et al. 1983; Farrar and Farrar 1986). Therefore, the half-life estimate of sucrose must have been dominated by the tracer ^13^C incorporation in those tissues that contained the bulk of the sucrose. As we sampled only near the end of the light period, a significant fraction of the sucrose in the leaf blades may have been vacuolar (Wagner et al. 1983), if the plants used a diurnal sucrose storage mechanism (eg Gerhardt et al. 1987). However, this factor would tend to increase the mean residence time of sucrose molecules in the pool (Farrar and Farrar 1986), which was not obvious in our data. Thus, the half-life of sucrose observed here was close to (or not much longer) than that of the “transport” sucrose pool (Farrar and Farrar 1986), which was defined to include all sucrose not stored in the vacuole of barley leaf blades. Additionally, we would not expect a strong effect of carbohydrate metabolism within sink tissue on the tracer dynamics at the whole-shoot level, since carbohydrate contents of that tissue (ie the leaf growth-and-differentiation zones, analyzed by Baca Cabrera et al. (2020) in the same experiment) accounted for only a small fraction of the total WSC in the shoot (<10%; Table S7). Taken together, the predominance of sucrose in the cytosol of parenchyma in leaf blades and sheaths, and the short transit times in the transport system linking the plant parts, likely combined to yield a half-life of whole-shoot sucrose that was very close to that of the “transport” pool of individual leaves (Borland and Farrar 1988).

### The key impact of increasing [CO_2_] on central carbohydrate metabolism was to increase sucrose cycling through the fructan pool

The fundamental effect of increasing [CO_2_] from 200 to 800 μmol mol^−1^ was to increase the flux of substrate to fructan synthesis (*F*_12_) by 2.8-fold per unit plant leaf area, the product of a 1.7-fold enhancement of substrate influx into the carbohydrate system (*F*_in_) and a 1.6-fold enhancement of the proportion of sucrose partitioned to fructan synthesis (Table 3), although the latter was not strictly statistically significant. At the same time, the flux of sucrose through futile cycling (sucrose hydrolysis/resynthesis) and partitioning to shoot growth and respiration plus export to roots remained relatively stable (see below). This, along with an unchanged half-life of all WSC pools, led to a strong fructan accumulation with [CO_2_].

Notwithstanding, the concentration of fructans in shoot biomass was already very high (35% of shoot biomass) even at 200 μmol mol^−1^ [CO_2_] (compare Tables 2 and 3, or Table S8). The generally high levels of fructan accumulation in this work likely resulted (at least partly) from a generally high level of assimilate availability for fructan storage owing to the high photosynthetically active radiation (800 µmol m^−2^ s^−1^ PPFD) maintained at canopy height throughout the 16-h-long photoperiod and simultaneous N limitation of growth. Such an N limitation was also reflected in generally low leaf elongation rates and reduced epidermal cell lengths in all treatments in the same experiment (Baca Cabrera et al. 2020). Beyond this, observed effects on carbohydrate metabolism were likely also influenced by an interaction between [CO_2_] and canopy N nutrition status as assessed by NNI (Lemaire et al. 2008). An analogous decrease of NNI with [CO_2_] has also been observed in grass-rich plant communities during the last century (Baca Cabrera et al. 2021) and in FACE experiments (Lüscher et al. 2004; see also Bassiouni et al. 2025) and was also indicated when the treatment effect on “dilution” of N by WSC was corrected for in the NNI analysis (Table 1). This decrease of NNI with [CO_2_] likely also caused the small reduction of whole-plant leaf area at 800 μmol mol^−1^ [CO_2_] relative to 200 μmol mol^−1^ [CO_2_]. Although the mechanisms underlying the decrease of vegetation N nutrition status with [CO_2_] are not fully understood (Gojon et al. 2023), the phenomenon is widespread (see also above) and decreased transpiration at high [CO_2_]—also observed in the present experiments (Baca Cabrera et al. 2020)—and associated reduced transpiration-driven advection of nitrate to the root systems (eg McGrath and Lobell 2013) may be connected with it.

Given the high fructan concentration even at 200 μmol mol^−1^ [CO_2_], the present data do not support a fundamental C balance penalty for fructan storage in this C_3_ grass at low [CO_2_], at least under the conditions of the experiment. On the other hand, the stimulation of fructan accumulation at 800 μmol mol^−1^ [CO_2_] agrees with observations of [CO_2_]-driven increased fructan levels in FACE experiments particularly in N limited conditions (Isopp et al. 2000). Remarkably, whole-shoot fructan concentration at 800 μmol mol^−1^ [CO_2_] was higher than any previous report for an intact system. In that context, it is notable that fructan concentrations of up to >70% of biomass were observed in detached illuminated leaves (Wagner et al. 1983), suggesting—in principle—a virtually nonlimiting capacity for fructan storage in future high [CO_2_] conditions.

The half-life of the fructan pool (∼7.7 d) was much longer than that in mature photosynthesizing leaf blades of the same cultivar of *L. perenne* grown with high or low N fertilizer supply (T_1/2_ 1.1 d or 2.6 d, respectively) in continuous light (Lattanzi et al. 2012) and equivalent to a mean residence time of C in the fructan pool of ∼11 d, close to the leaf appearance interval observed by Baca Cabrera et al. (2020) in the same experiments. This residence time corresponded to about one-third of the average leaf life span of *L. perenne* (Schleip et al. 2013). Although these relationships do not prove that fructan storage was constrained by leaf life span directly, we cannot rule out that developmental cues associated with leaf senescence were perhaps involved in the control of fructan storage and mobilization. Indeed, Volenec (1986) already associated fructan dynamics in leaf sheaths to leaf turnover.

### Metabolic resilience of the carbohydrate system

Remarkably, the summed proportion of sucrose cycling through fructans and futile cycling (hydrolysis/resynthesis) (Table 3) was insensitive to [CO_2_]. At the same time the proportion of sucrose use in growth and maintenance (including export to roots) was practically constant. Yet, partitioning of the gross flux of sucrose use between futile cycling and fructan synthesis changed considerably with [CO_2_], with the rate of sucrose consumption in hydrolysis about 2 times that in fructan synthesis at 200 μmol mol^−1^ [CO_2_], but similar at 400 and 800 μmol mol^−1^ [CO_2_] (Table 3). Futile cycling of sucrose has been implied as a key factor in central carbohydrate metabolism in stabilizing sucrose concentration and being involved in partitioning of sucrose between export and carbohydrate storage; sugar transport between cytosol, vacuole, and apoplast; and feedback control of photosynthesis (eg Huber 1989; Geigenberger and Stitt 1991; Nguyen-Quoc and Foyer 2001; Weiszmann et al. 2018). As it stabilizes sucrose concentration, futile cycling should also interact with sucrose availability for fructan synthesis or export/partitioning to growth and respiration, the main sinks for sucrose. Probably, such a mechanism would depend on the presence of the interacting mechanisms in the same tissue, information which is not available for this work. Yet, it is well known that sucrose futile cycling is common and active in photosynthesizing leaves of dicots and grasses (Wagner et al. 1986; Lemoine 2000), including in *L. perenne* (Lattanzi et al. 2012; but see Cairns and Gallagher 2004) as well as in sink tissue (eg Nguyen-Quoc and Foyer 2001). The estimates of futile cycling in the present study were comparable in magnitude to estimates for leaves of *Arabidopsis* by Nägele et al. (2010), soybean and tobacco by Huber (1989), and cotyledons of germinating *Ricinus communis* by Geigenberger and Stitt (1991). It has been proposed that leaf sucrose futile cycling should increase as photosynthesis acclimates to elevated [CO_2_] (Moore et al. 1999). However, our results do not support this prediction at shoot level, although—again—we cannot rule out that a possible enhancement of sucrose futile cycling in source tissue (leaf blades) was offset by decreased cycling in sink tissue (eg fructan storing leaf sheaths). Indeed, Lattanzi et al. (2012) observed much greater sucrose futile cycling in leaf blades in continuous light.

Perhaps most significantly in this work, the simultaneous presence of active futile cycling of sucrose and sucrose cycling through the fructan pool together constitutes a strong buffering mechanism for sucrose availability for growth and maintenance activities throughout the plant when photosynthesis fluctuates naturally or under stress or disturbance. Given the half-lives of the sugar pools involved, their size, and the magnitude of the fluxes, the 2 activities may combine to form a resilience mechanism which enables buffering of sucrose availability over timescales of less than 1 hour up to several weeks.

### Conclusions

In our opinion, the most important finding of this work is that it highlights the mechanism by which fructan metabolism is involved in sucrose regeneration at whole-shoot level per se and in response to variation of [CO_2_] from 200 to 800 µmol mol^−1^. That involvement was most substantial, as the rate of sucrose regeneration from the products of fructan hydrolysis (estimated as *F*_24_, Table 3), was similar in magnitude to the concurrent rate of sucrose neo-synthesis from products of photosynthesis (*F*_in_) at 400 μmol mol^−1^ [CO_2_]. At 200 and 800 µmol mol^−1^ [CO_2_], it was equivalent to 65% and 113% of sucrose neo-synthesis, respectively. This result also explains why the time taken for 50% labelling of the whole-shoot sucrose pool (∼2 d) was more than one order of magnitude higher than the half-life of this pool (∼2.4 h). A constitutive involvement of the fructan store in sucrose resynthesis would also explain the large half-life of the total “metabolic” pool supplying synthesis of structural biomass (T_1/2_ ∼3–5 d, Ostler et al. 2016) or autotrophic respiration of a grassland ecosystem (T_1/2_ ∼2.6 d, Gamnitzer et al. 2009) or shoot and root respiration in *L. perenne* (T_1/2_ ∼2 d, Lehmeier et al. 2008), testifying to the virtual universality of this resilience feature (at least) in this fructan storing C_3_ grass.

As a perspective we (i) advocate future studies of the co-occurrence (yes or no) of sucrose cycling via futile cycling and fructan metabolism in the same tissue (or cells), and of putative interactions between sucrose futile cycling and sucrose cycling through the fructan pool, controlling the gradient of sucrose between the cytosol and vacuole (or cytosol and phloem), (ii) suggest similar studies comparing the involvement and control of NSC stores (and associated sucrose cycling) in different plant functional types, also in relation to different types of NSC stores (eg fructan vs starch vs sucrose) subjected to different stress scenarios. Naturally, such works will also require the design of storage-form-specific compartmental models for starch- and/or sucrose-storing species. Lastly (iii), NSC stores should be more generally considered (eg in plant growth models) as constitutively turning over (as opposed to induced) components that buffer continuously carbohydrate supply and demand at timescales of hours to several weeks or longer.

## Materials and methods

### Experimental design, treatments, and growth conditions

The experiment had a factorial design with 3 [CO_2_] levels (200, 400, or 800 μmol mol^−1^) as the factor (see below). The work made use of the plant growth chamber–based ^13^CO_2_/^12^CO_2_ gas exchange-facility recently presented by Zhu et al. (2025) in a study of the long-term precision and accuracy of the mesocosm-scale ^13^CO_2_/^12^CO_2_-labelling facility in the same work. This facility included 4 chambers, which implied that sequential experimental runs were needed for chamber-scale replication of the experiment. This was done in such a way that the 200 and 800 μmol mol^−1^ [CO_2_] treatments were executed in parallel with the “reference” 400 μmol mol^−1^ [CO_2_] treatment in successive experimental runs (Baca Cabrera et al. 2020). Thus, 2 chamber-scale replicates of the 200 and 400 μmol mol^−1^ [CO_2_] treatments were tested in the first run, and 2 replicates each of the 400 and 800 μmol mol^−1^ [CO_2_] treatments in the second run.

Details of the plant material and growth conditions have been presented by Baca Cabrera et al. (2020) in a study of mechanisms controlling leaf elongation rate, and Zhu et al. (2025). Briefly, the study material consisted of perennial ryegrass (*L. perenne* L., var Acento) plants grown singly from seed in plastic pots (350 mm height, 50 mm diameter) filled with 800 g of washed quartz sand (0.3 to 0.8 mm grain size). Pots were arranged at a density of 383 plants m^−2^ in plastic containers (770 × 560 × 300 mm), and 2 of such containers placed in each growth chamber (PGR15, Conviron, Winnipeg, Canada). In all chambers, air temperature and relative humidity (RH) were controlled at 20/16 °C and 50%/75% during the 16/8-h day/night periods until the end of the experiment, on d 74 after first imbibition of seeds. A photosynthetic photon flux density (PPFD) of 800 µmol m^−2^ s^−1^ was maintained at canopy height during the light period. A Hoagland-type nutrient solution with two-thirds-strength nitrate-N concentration relative to standard and nominal concentrations of all the other nutrients was supplied every 6 h by flooding the containers for 9 min followed by draining by gravity (Baca Cabrera et al. 2020).

### CO_2_ control and ^13^CO_2_/^12^CO_2_ labelling

Instrumentation and protocols for controlling the concentration and isotopic composition (δ^13^C_CO2_) of CO_2_ inside the plant growth chambers were described by Zhu et al. (2025). Briefly, all chambers were operated individually as open gas exchange systems, with air supply to the chambers controlled such that the [CO_2_] between the inlet and outlet of the chamber decreased by not more than 10% to 20%, while keeping the [CO_2_] inside the highly ventilated chambers close (ie within 2%) to the nominal (target) value in all [CO_2_] treatments. Air for the chambers was mixed from dry, CO_2_-free air and tank CO_2_ using mass flow controllers. [CO_2_] and water vapor concentration at the inlet and outlet of each growth chamber were measured every 30 min by an infrared gas analyzer (IRGA, Li-840, Li-Cor, Lincoln, Nebraska, United States).

[CO_2_] treatments were installed on d 12 following first imbibition of seeds and continued until the end of the experiment on d 74. Parallel chambers within a given [CO_2_] treatment were supplied with a different δ^13^C of CO_2_ (δ^13^C_CO2_), that is, a relatively ^13^C-depleted CO_2_ source (δ^13^C ∼ −43.5‰) derived from industrial processes (Linde AG, Unterschleissheim, Germany) or a ^13^C-enriched CO_2_ (δ^13^C ∼ −5.6‰) originating from a mineral water source (CARBO Kohlensäurewerke, Bad Hönningen, Germany).

On d 66 in both experimental runs, when plants in all treatments had established dense canopies (leaf area index >6), ^13^C-labelling of the entire photosynthetic C flux of all canopies was initiated by switching the CO_2_ sources as in Schnyder et al. (2003). Thus, a growth chamber that had received the ^13^C-enriched CO_2_ henceforth received the ^13^C-depleted CO_2_, and vice versa. The switch occurred during the night of d 65 to d 66, not later than 4 h before the onset of the next light period. This procedure ensured that the isotopically “old” (ie “pre-switch”) CO_2_ had been virtually completely replaced by the “new” CO_2_ inside the chambers at the beginning of the first labelling light period. In all experiments, the continuous labelling period lasted for a total duration of 7 d.

To minimize contamination of cabinet air with extraneous CO_2_, (i) a slight atmospheric overpressure was maintained inside the chambers by restricting air flow at the chamber outlet, (ii) air locks were installed in chamber doors to minimize air incursion when chambers had to be opened, and (iii) activities inside the chambers were kept at a minimum within the restrictions of the experimental plan. Nevertheless, we determined a small isotopic contamination effect of daytime air incursion on the isotopic composition of biomass and WSC samples: this isotopic contamination amounted to approximately 3.3% for the different biomass and WSC components and was independent of the [CO_2_] treatments (Zhu et al. 2025).

### Sampling

Starting on d 65 we sampled plants for subsequent determinations of C labelling kinetics in carbohydrates. These harvests occurred always at the beginning of the dark period and first took place just prior to the switch of δ^13^C_CO2_ and then after the first, second, third, fourth, and seventh photoperiod of labelling. On each date, 2 replicate samples were collected from each growth chamber, with 3 randomly selected plants accounting for/constituting one replicate. Gaps in the canopies resulting from collection of plants were immediately filled with plants taken from the periphery of the containers.

Sampled plants were removed from their pots, their roots washed to free them of sand, and the plants dissected at the shoot–root interface. Shoots and roots were separately combined by replicate, and fresh weight was determined. Samples were then shock-frozen in liquid N_2_ and stored at −18 °C until later freeze-drying for 72 h. Subsequently, plant material was ground to a fine powder in a ball mill (Mixer mill MM 400, Retsch, Haan, Germany) with stainless steel beads in stainless steel grinding jars and thereafter stored again at −18 °C until further use (Zhu et al. 2025).

### Leaf area

Eight randomly selected plants from each growth chamber were sampled on d 64—1 d prior to initiation of labelling—to measure total leaf area per plant following the protocol of Baca Cabrera et al. (2020). The leaf area of plants collected later, ie during labelling, was then estimated using an allometric model derived from the above plant morphology dataset. Leaf area index (LAI, m^2^ m^−2^) of the plant canopies was extrapolated from these measurements as LAI = leaf area per plant × number of plants per unit ground area.

### Water-soluble carbohydrate extraction, separation, and quantification

WSC fractions (sucrose, fructans, glucose, and fructose) were extracted from shoot samples and separated using the procedures described by Zhu et al. (2025; see also Schnyder and de Visser 1999 and Gebbing and Schnyder 2001). Briefly, aliquots of 200 mg of milled sample material were weighed into 2-mL capped Eppendorf tubes and topped off with 1.8 mL deionized water. Samples were briefly vortexed, held in a water bath at 93 °C for 10 min, shaken for 45 min at room temperature, and centrifuged at 9,500 *g* for 15 min. The supernatant, which contained the dissolved WSC, was passed through nylon-membrane filters with a pore size of 0.45 µm and then stored in clean 2-mL capped Eppendorf tubes at −18 °C. WSC fractions (sucrose, fructans, glucose, and fructose) were separated, quantified, and collected using an HPLC system similar to that of Gebbing and Schnyder (2001): 0.2-mL aliquots of the filtered supernatant were passed through a guard column (Shodex KS-LG, Showa Denko, Tokyo, Japan) and preparative column (Shodex Sugar KS2002, 300 × 20 mm, Showa Denko, Tokyo, Japan) held at 50 °C, with HPLC-grade water as the eluent, at a flow rate of 0.75 mL min^−1^. This procedure resulted in a clean separation of the individual carbohydrate components (Zhu et al. 2025; see also Table S2). The WSC were detected by refractive index measurement (Shodex RI-101, Showa Denko, Tokyo, Japan) and concentrations quantified by comparing sample peak areas against reference calibration curves of pure and mixed standards of analytical grade inulin, sucrose, glucose, and fructose (all from Merck, Darmstadt, Germany). Knowing the time when the individual carbohydrates eluted from the preparative column, we collected fractions of fructans, sucrose, glucose, and fructose individually in test tubes. The total WSC concentration in the shoot was obtained as the sum of the concentrations of sucrose, fructans, glucose, and fructose.

### Elemental and isotope analysis

The C and N contents of shoot and root samples (as well as the δ^13^C of these biomass samples, not reported here) were determined as in Baca Cabrera et al. (2021). For this, the stored samples were thawed, re-dried at 40 °C for 24 h, and stored in exsiccator vessels. Then 0.75 ± 0.05-mg aliquots of shoot and root material were packed into tin cups (IVA Analysentechnik, Meerbusch, Germany) and combusted in an elemental analyzer (Carlo Erba NA 1110, Carlo Erba Instruments, Milan, Italy) interfaced (Conflo III, Finnigan MAT, Bremen, Germany) to a continuous-flow isotope-ratio mass spectrometer (CF-IRMS; Delta Plus, Finnigan MAT, Bremen, Germany) that measured concentrations of C and N and δ^13^C of samples. A solid internal laboratory standard (SILS, fine ground wheat flour) was measured as a reference after every 10th sample to correct for eventual instrument drift. All samples and SILS were measured against a laboratory working standard CO_2_ gas, which was previously calibrated against a secondary isotope standard (IAEA-CH6; accuracy of calibration ±0.06‰ SD). The error (SD) of repeated measurements of SILS was <0.2‰.

Aliquots comprising approximately 0.70 mg of the different carbohydrates were transferred to tin cups, dried at 60 °C for 24 h, and then analyzed for their δ^13^C using a CF-IRMS as described above.

### The fraction of tracer in carbohydrate

Tracer incorporation in carbohydrates was followed via the time course of the fraction of unlabelled C (ƒ_unlab_) in the different carbohydrates (sucrose, fructans, glucose, and fructose) during the 7-d-long continuous labelling periods as in Schnyder et al. (2003) and Lattanzi et al. (2012). ƒ_unlab_ represents the fraction of “old” C in a carbohydrate pool, that is, the C that was assimilated prior to the switch of the isotopic composition of chamber CO_2_. Following the switch, the old C was gradually replaced by a fraction of “new” (ie “labelled’) C (ƒ_lab_, with ƒ_lab_ = 1 – ƒ_unlab_), assimilated from the isotopically “new” CO_2_ in the chamber. ƒ_unlab_ was determined as:


(1) $$f_{\mathrm{unlab}}=(\delta^{13}C_{\mathrm{sample}}\text{–}\delta^{13}C_{\mathrm{new}})/(\delta^{13}C_{\mathrm{old}}\text{–}\delta^{13}C_{\mathrm{new}}),$$

with *δ*^13^C_sample_ denoting the *δ*^13^C of a certain carbohydrate species (fructans, sucrose, glucose, or fructose, as appropriate) sampled on a given day in a given [CO_2_] treatment, and δ^13^C_old_ and δ^13^C_new_ denoting the δ^13^C of the same carbohydrate extracted from plants that were harvested immediately before the switch of δ^13^C_CO2_ in the same [CO_2_] treatment. In this way, we obtained time courses of *f*_unlab_ for both the switches from the ^13^C-enriched to the ^13^C-depleted CO_2_, and from the ^13^C-depleted to the ^13^C-enriched CO_2_ (Schnyder et al. 2003).

### Compartmental analysis of tracer time course

We used the same 4-pool compartmental model variants as Lattanzi et al. (2012) (Table S1) based on the same set of differential equations and implemented in the same software (ModelMaker, Cherwel Scientific, United Kingdom) to analyze the time course of ƒ_unlab_ in the different carbohydrate pools at the whole-shoot scale. For every carbohydrate pool *Q*_i_ (with *Q*_1_, *Q*_2_, *Q*_3_, and *Q*_4_ representing the size of the sucrose, fructan, glucose, and fructose pools, respectively), it is assumed that *dQ*_i_/*dt* = 0, which implies that the sum of all fluxes of C into and out of *Q*_i_ has the same magnitude. Our assumptions also implied that a given flux out of a donor pool *i* to a receiving pool *j* (*F*_ij_) is the product of a rate constant (*k*_ij_) and the size of the donor pool (*Q*_i_) (*F*_ij_ = *k*_ij_  *Q*_i_). *j* = 0 denotes a flux leaving the 4-pool system and reflects sucrose consumption in all growth and maintenance processes in the entire plant, including export to roots. The half-life of a pool (T_1/2 *Q*i_) is T_1/2 *Q*i_ = ln(2)/*k*_out_i_ [or ln(2)/(Σ_j_ k_ij_)], with *k*_out_i_ the sum of the rate constants for all fluxes leaving pool *Q*_i_.

The “constrained 4-pool model” (displayed in Fig. 1b) dictated a 1:1 fructose:glucose ratio in the resynthesis of sucrose and does not allow for fructose–glucose interconverting reactions (compare with Table S1). This model best reflected the tracer data in the present experiment (see Table S3) and is described by the following set of differential equations. In these, the change in the amount of unlabelled C in pool *i* with labelling time *t* is given by the sum of all fluxes of unlabelled C into that pool minus the sum of all fluxes of unlabelled C leaving the pool; thus:


(2) $$\begin{array}{rll} df_{\mathrm{unlab}\;Q1} & (t)/dt(t)\times Q_{1}=f_{\mathrm{unlab}\;\mathrm{in}}(t)\times F_{\mathrm{in}}+ & f_{\mathrm{unlab}\;Q3}(t)k_{31}Q_{3}\\ + & f_{\mathrm{unlab}\;Q4}(t)k_{41}Q_{4}\text{–}f_{\mathrm{unlab}\;Q1}(t)\times (k_{10}+k_{12}+k_{13}+k_{14})Q_{1}, \end{array}$$

(3) $$df_{\mathrm{unlab}\;Q2}(t)/dt(t)\times Q_{2}=f_{\mathrm{unlab}\;Q1}(t)\times k_{12}Q_{1}\text{–}f_{\mathrm{unlab}\;Q2}(t)\times k_{24}Q_{2},$$

(4) $$df_{\mathrm{unlab}\;Q3}(t)/dt(t)\times Q_{3}=f_{\mathrm{unlab}\;Q1}(t)\times k_{13}Q_{1}\text{–}f_{\mathrm{unlab}\;Q3}(t)\times k_{31}Q_{3},$$

(5) $$df_{\mathrm{unlab}\;Q4}(t)/dt(t)\times Q_{4}=f_{\mathrm{unlab}\;Q1}(t)\times k_{14}Q_{1}+f_{\mathrm{unlab}\;Q2}(t)\times k_{24}Q_{2}\text{–}f_{\mathrm{unlab}\;Q4}(t)\times k_{41}Q_{4};$$

*f*_unlab Qi_ (*t*) denotes the fraction of unlabelled C in pool *i* at time *t*, *F*_in_ the flux into the system (Fig. 1), and *f*_unlab in_ (*t*) the fraction of unlabelled C in *F*_in_ with *f*_unlab in_ (*t*) = 1 before the switch of δ^13^C_CO2_ (ie at *t* < 0) and *f*_unlab in_ (*t*) = 0 at and after the switch (*t* ≥ 0).

Based on Eqs. 2–5, the rate constants *k*_10_, *k*_12_, and *k*_14_ were optimized in such a way that *f*_unlab Qi_ (*t*) fitted best the observed *f*_unlab Qi_ (*t*) for the tracer kinetics of sucrose (*Q*_1_), fructan (*Q*_2_), glucose (*Q*_3_), and fructose (*Q*_4_) pools. In this, we constrained the model with the pool size ratios *Q*_i_/*Q*_j_ as observed in the experiments.

Fluxes into the system and between pools—from donor to receiving pool—were obtained thus (as in Lattanzi et al. 2012):


(6) $$F_{\mathrm{in}}=F_{\mathrm{out}}=k_{10}Q_{1},$$

(7) $$F_{12}=F_{24}=k_{12}Q_{1}=k_{24}Q_{2},$$

(8) $$F_{13}=F_{31}=F_{41}=k_{13}Q_{1}=k_{31}Q_{3}=k_{41}Q_{4},$$

(9) $$F_{14}+F_{24}=k_{14}Q_{1}+k_{24}Q_{2}=k_{41}Q_{4},$$

with *F*_in_ the photosynthetically neo-synthesized influx of C into the sucrose pool, the entry point of the system, and *F*_out_ the flux of C out of the carbohydrate system via the sucrose pool. Equating *F*_31_ = *F*_41_ in Eq. 8 reflected the stochiometric constraint of a 1:1 fructose:glucose ratio in the resynthesis of sucrose in the “constrained” model variant (see above).

The mean residence time (τ) of C in the system was calculated as:


(10) $$\tau =(Q_{1}+Q_{2}+Q_{3}+Q_{4})/F_{\mathrm{in}},$$

As in Lattanzi et al. (2012), the half-life (*T*_1/2_) of the different pools was obtained as:


(11) $$T_{1/2}(Q_{1})=\ln\;(2)/(k_{10}+k_{12}+k_{13}+k_{14}),$$

(12) $$T_{1/2}(Q_{2})=\ln\;(2)/k_{24},$$

(13) $$T_{1/2}(Q_{3})=\ln(2)/k_{31},$$

(14) $$T_{1/2}(Q_{4})=\ln\;(2)/k_{41},$$

### N nutrition index

The NNI is a widely used crop ecophysiological parameter that quantifies the N status of crop stands (Lemaire et al. 2008). Accordingly, the NNI is determined as the ratio of the actual (measured) N concentration in dry-shoot biomass (*N*_a_; expressed as %N, w/w × 100) relative to the “critical” (ie minimum for maximum growth) N concentration (*N*_c_) of a plant stand with the same dry-shoot biomass (expressed in metric ton per hectare; *W*, t ha^−1^):


(15) $$NNI=N_{a}/N_{c}.$$

Stands with an NNI = 1 have just enough N for maximum growth; stands with NNI < 1 display N-limited growth.

The critical N concentration is dependent on *W* according to a negative power function (“dilution curve”) as:


(16) $$N_{c}=aW^{-b}$$

for stands with a *W* > 1 t ha^−1^. Coefficient *a* (4.8 for temperate grasses) represents *N*_c_ at *W* = 1 t ha^−1^, and coefficient *b* (0.32) is dimensionless. NNI was calculated from *N*_a_ of shoot biomass as measured on d 63; *N*_c_ was determined with Eq. 16 based on *W* estimated from dry-shoot biomass per plant and plant number per unit area sampled on d 63.

To account for an eventual [CO_2_]-dependent dilution effect of N (and hence NNI) by WSC, we also calculated a “corrected NNI” (NNI_corr_) based on a recalculation of *N*_a_ and *W* using the assumption that whole-shoot WSC concentration was 20% (w/w) in all [CO_2_] treatments.

### Statistics

One-way ANOVA with Tukey's HSD post hoc tests for pairwise comparisons was employed to explore the effect of CO_2_ treatments on leaf area, C mass of plants, shoots and roots, total WSC contents, and individual WSC components. All statistical analyses were conducted in R version 3.6.1 (R Core Team 2019) and RStudio version 1.1.383 (Rstudio Team 2016). The R packages ggplot2 (Wickham 2016) and agricolae (de Mendiburu 2017) were used for data plotting and ANOVA, respectively. The number of replicates varied between measured parameters and treatments and is indicated in the figure legends and table captions.

For statistical comparison of model parameters between CO_2_ treatments, CO_2_ concentration was treated as a discrete factor with 3 levels (200, 400, and 800 μmol mol^−1^). For each parameter of the compartmental model, a single estimate and its standard error (SE) were obtained per treatment by fitting the compartmental model independently to the tracer data. Because conventional ANOVA requires multiple independent replicate estimates per treatment, it was not applicable. Instead, we performed pairwise 2-sample *z*-tests to compare parameter estimates between CO_2_ levels. The test statistic was calculated as:


(17) $$z=\frac{\left|\theta_{1}-\theta_{2}\right|}{\sqrt{SE_{1}^{2}+SE_{2}^{2}}},$$

where θ_1_, SE_1_, θ_2_, SE_2_ are the estimate and SE from the 2 treatments being compared. *P* values were obtained from the standard normal distribution, and differences with *P* < 0.05 were considered significant.

The same *z*-test formula was applied, assuming independence, for pairwise comparisons among the 3 sucrose partitioning fractions within the same CO_2_ treatment. Because these 3 fractions sum to 1, they are correlated; therefore, these within-treatment comparisons are approximate and serve as an exploratory guide.

To select the best compartmental model among the variants tested (see Table S1), we used the BIC. It was calculated as:


(18) $$\mathrm{BIC}=\mathrm{nln}\left(\frac{\mathrm{SSE}}{n}\right)+\mathrm{kln}(n),$$

where *n* is the number of data points, *SSE* is the residual sum of squares, and *k* is the number of model parameters. This expression simplifies the standard BIC by omitting a constant term that is identical across all models compared; therefore, only differences in BIC matter. Lower BIC values (more negative) indicate a better fit after penalizing model complexity.

Language editing: The manuscript was professionally edited for grammar and clarity. No artificial intelligence tools were used for data generation, analysis, or figure creation.

## Supplementary Material

kiag381_Supplementary_Data

## Data Availability

The datasets that support the findings of this study are available from the corresponding author on reasonable request.
