# Supplementary material for: Extensive sucrose cycling through the fructan pool of a C3 grass across a 200 to 800 μmol mol−1 atmospheric CO2 gradient
Source: Plant Physiol. 2026 Jun 16;201(4):kiag381. doi: 10.1093/plphys/kiag381 (PMC13525200; doi:10.1093/plphys/kiag381)
Supplement: Supplementary file 1 [file kiag381_supplementary_data.docx]

**Supplementary material**

Article title: **Extensive sucrose cycling through the fructan pool of a C_3_ grass across a 200 to 800 μmol mol^-1^ atmospheric CO_2_ gradient**

The following Supporting data are available for this article:

**Supporting Table S1** Structure and specification of alternative 4-pool models of central carbohydrate metabolism.

**Supporting Table S2** Results of a one-way ANOVA testing the chamber effect on plant, shoot and root biomass, leaf area per plant, and whole-shoot WSC, fructan, sucrose, glucose and fructose contents per unit leaf area.

**Supporting Table S3** Results of a one-way ANOVA testing the effect of atmospheric CO_2_ concentration on morphophysiological parameters displayed in Tables 1 and 2 and Fig 2.

**Supporting Table S4** δ^13^C of reference carbohydrates measured pure or after mixing and separation by HPLC.

**Supporting Table S5** Optimized parameter values and statistical properties of alternative 4-pool models of central carbohydrate metabolism in leaves of *Lolium perenne* L*.*

**Supporting Table S5‑1.** Pairwise comparisons of sucrose partitioning fractions (Table 3) within each CO₂ treatment.

**Supporting Table S5‑2.** Pairwise *P*‑values from two‑sample z‑tests comparing each parameter in Table 3 among three CO_2_ concentrations (200, 400 and 800 μmol mol^-1^).

**Supporting Table S6** Results of a one-way ANOVA testing the effect of time on carbohydrate content per unit leaf area at each CO_2_ concentration during dynamic labelling.

**Supporting Table S7** Shoot and root growth rates of plants during the final three weeks of the experiment and the fraction of WSC in the leaf growth and differentiation zones (LGDZ) relative to whole shoot biomass.

**Supporting Table S8** Water-soluble carbohydrate concentrations (WSC: sucrose, fructan, glucose and fructose) in whole shoot biomass of perennial ryegrass (*Lolium perenne* L) as affected by atmospheric CO_2_ concentration during growth.

**Supporting Table S1** Structure and specification of alternative 4-pool models of central carbohydrate metabolism in the shoot of *Lolium perenne*, following Lattanzi et al., 2012. Pools: 1, Sucrose; 2, Fructan; 3, Glucose; 4, Fructose. Arrows refer to rate constants (see Materials and Methods). For biochemical details see main text and Lattanzi et al. (2012).

| **Model name** | **Structure** | **Specification** |
| --- | --- | --- |
| **Free** |  | 4-pool model, which does not contain stoichiometric constraints for glucose and fructose use in sucrose resynthesis, and allows fructose-glucose interconverting  reactions |
| **Free without** |  | 4-pool model, which does not contain the 1:1 stoichiometric constraint for glucose and fructose use in sucrose resynthesis, but also does not allow for fructose-glucose interconverting  reactions |
| **Constrained** |  | Constrained 4-pool model which dictates a 1:1 fructose:glucose ratio in the (re-)synthesis of sucrose and does not allow for fructose-glucose interconverting reactions |

**Supporting Table S2** Results of a one-way ANOVA testing the chamber effect on plant, shoot and root biomass, leaf area per plant, and whole-shoot WSC, fructan, sucrose, glucose and fructose contents per unit leaf area.

| **CO_2_ concentration** | **Parameter** | ***F*-value** | ***P*-value** |
| --- | --- | --- | --- |
| 200 μmol mol^-1^ |  |  |  |
|  | Plant biomass | 2.82 | 0.11 |
|  | Shoot biomass | 3.66 | 0.69 |
|  | Root biomass | 0.61 | 0.44 |
|  | Leaf area per plant | 3.54 | 0.07 |
|  | WSC | 0.04 | 0.85 |
|  | Fructan | 0.16 | 0.69 |
|  | Sucrose | 0.11 | 0.74 |
|  | Glucose | 0.43 | 0.52 |
|  | Fructose | 0.08 | 0.78 |
| 400 μmol mol^-1^ |  |  |  |
|  | Plant biomass | 1.00 | 0.32 |
|  | Shoot biomass | 0.78 | 0.39 |
|  | Root biomass | 0.05 | 0.83 |
|  | Leaf area per plant | 0.23 | 0.64 |
|  | WSC | 0.38 | 0.54 |
|  | Fructan | 0.35 | 0.56 |
|  | Sucrose | 0.84 | 0.36 |
|  | Glucose | 0.08 | 0.77 |
|  | Fructose | 0.01 | 0.94 |
| 800 μmol mol^-1^ |  |  |  |
|  | Plant biomass | 0.90 | 0.35 |
|  | Shoot biomass | 0.78 | 0.39 |
|  | Root biomass | 0.16 | 0.69 |
|  | Leaf area per plant | 0.87 | 0.36 |
|  | WSC | 0.26 | 0.61 |
|  | Fructan | 0.23 | 0.64 |
|  | Sucrose | 0.13 | 0.73 |
|  | Glucose | 0.37 | 0.55 |
|  | Fructose | 1.4 | 0.25 |

**Supporting Table S3** Results of a one-way ANOVA testing the effect of atmospheric CO_2_ concentration on morphophysiological parameters displayed in Tables 1 and 2 and Fig 1.

| **Parameter** | ***F*-value** | ***P*-value** |
| --- | --- | --- |
| Plant-carbon (g plant^-1^) | 39.88 | <0.001 |
| Shoot-carbon (g plant^-1^) | 32.67 | <0.001 |
| Root-carbon (g plant^-1^) | 33.24 | <0.001 |
| Shoot : root ratio | 1.99 | 0.14 |
| NNI | 109.7 | <0.001 |
| Leaf area per plant | 4.67 | 0.01 |
| WSC (g plant^-1^) | 34.47 | <0.001 |
| WSC (mg g^-1^) | 15.34 | <0.001 |
| WSC (g m^-2^) | 78.2 | <0.001 |
| Fructan (g m^-2^) | 71.2 | <0.001 |
| Sucrose (g m^-2^) | 26.9 | <0.001 |
| Glucose (g m^-2^) | 15.9 | <0.001 |
| Fructose (g m^-2^) | 52.9 | <0.001 |

**Supporting Table S4** δ^13^C of reference carbohydrates measured pure or after mixing and separation by HPLC.

| Group | Fructan | Sucrose | Glucose | Fructose |
| --- | --- | --- | --- | --- |
|  | δ^13^C (‰) | | | |
| Pure | -25.14 ± 0.03 | -25.32 ± 0.04 | -10.61 ± 0.14 | -25.25 ± 0.09 |
| Mixture | -25.20 ± 0.20 | -24.94 ± 0.37 | -11.00 ± 0.87 | -25.02 ± 0.40 |

The δ^13^C of analytical grade inulin-fructan, sucrose, glucose and fructose (all from Merck, Darmstadt, Germany) was determined either as pure material or after mixing and separation by HPLC. Note the distinctly less negative δ^13^C of the glucose reference material (typical of a C_4_ origin) in comparison with the δ^13^C of fructan, sucrose and fructose, all of which have a C_3_ origin. The mixed WSC sample consisted of a 80 : 5 : 5 : 4 (wt : wt : wt : wt) mixture of fructan, sucrose, glucose and fructose, similar to the typical composition of a WSC sample extracted from shoots of plants grown at a [CO_2_] of 400 μmol mol^-1^. Data shown are means ± SD (*n* = 2-3). The δ^13^C of individual carbohydrates was not significantly different between pure and mixture groups (*P* > 0.05).

**Supporting Table S5** Optimized parameter values and statistical properties of alternative 4-pool models of central carbohydrate metabolism in leaves of *Lolium perenne* L*.*

|  | **CO_2_ concentration (μmol mol^-1^)** | | | | | | | | | | |
| --- | --- | --- | --- | --- | --- | --- | --- | --- | --- | --- | --- |
|  | 200 | | |  | 400 | | |  | 800 | | |
|  | **Model name** | | | | | | | | | | |
|  | Free | Free without | Constrained |  | Free | Free without | Constrained |  | Free | Free without | Constrained |
|  | **Rate constant (SE), h^-1^** | | | | | | | | | | |
| *k*_10_ | **0.059 (0.003)** | **0.060 (0.003)** | **0.062 (0.002)** |  | **0.060 (0.004)** | **0.064 (0.004)** | **0.065 (0.003)** |  | **0.050  (0.008)** | **0.050 (0.007)** | **0.056 (0.007)** |
| *k*_12_ | **0.039 (0.003)** | **0.039 (0.003)** | **0.039 (0.003)** |  | **0.050 (0.006)** | **0.054 (0.005)** | **0.055 (0.005)** |  | **0.050  (0.014)** | **0.050 (0.012)** | **0.063 (0.014)** |
| *k*_13_ | **0.71^ns^ (1.86)** | **0.22^ns^ (0.12)** | 0.12 (0.01) |  | **1.37^ns^  (11.80)** | **0.13^ns^ (0.07)** | 0.11 (0.01) |  | **0.90^ns^  (12.25)** | **2.30^ns^ (60.40)** | 0.13  (0.02) |
| *k*_14_ | **0.08^ns^ (0.21)** | **0.08**  **(0.01)** | **0.08**  **(0.01)** |  | **0.05**  **(0.11)** | **0.05**  **(0.01)** | **0.05**  **(0.01)** |  | **0.05  (0.33)** | **0.05**  **(0.01)** | **0.06**  **(0.02)** |
| *k*_24_ | 0.004 (0.0003) | 0.004 (0.0003) | 0.004  (0.0003) |  | 0.0035 (0.0004) | 0.0038 (0.0004) | 0.0038 (0.0003) |  | 0.0031 (0.0008) | 0.0031 (0.0008) | 0.0039 (0.0008) |
| *k*_31_ | **1.18^ns^ (3.23)** | 0.50**^ns^** (0.54) | 0.19 (0.02) |  | **2.60^ns^  (22.78)** | 0.27**^ns^** (0.14) | 0.21 (0.02) |  | **1.90^ns^  (23.40)** | 4.47**^ns^** (117.42) | 0.24  (0.05) |
| *k*_34_ | **0.002^ns^ (0.359)** |  |  |  | **0.002^ns^ (0.211)** |  |  |  | **0.004^ns^  (0.694)** |  |  |
| *k*_41_ | 0.16**^ns^** (4.38) | 0.18 (0.01) | 0.18 (0.02) |  | 0.22**^ns^**  (24.82) | 0.16 (0.01) | 0.16 (0.01) |  | 0.03**^ns^**  (22.67) | 0.12 (0.02) | 0.16 (0.03) |
| *k*_43_ | 0.03**^ns^** (4.38) |  |  |  | -0.07**^ns^**  (24.82) |  |  |  | 0.10**^ns^**  (22.67) |  |  |
|  | **Half-lives (SE), h** | | | | | | | | | | |
| Sucrose | 0.8 (1.7) | 1.8 (0.6) | 2.4 (0.2) |  | 0.5 (3.5) | 2.3 (0.5) | 2.5 (0.1) |  | 0.7  (7.7) | 0.3 (7.0) | 2.3 (0.4) |
| Fructan | 196 (17) | 195  (15) | 191  (15) |  | 200  (24) | 184 (18) | 182 (16) |  | 224 (61) | 224 (53) | 179 (39) |
| Glucose | 0.6 (1.6) | 2.0 (1.1) | 3.7 (0.3) |  | 0.3 (2.3) | 2.6 (1.3) | 3.3 (0.3) |  | 0.4  (5.0) | 0.2 (4.1) | 2.9 (0.5) |
| Fructose | 3.8 (3.9) | 3.8 (0.3) | 3.8 (0.3) |  | 4.6  (7.0) | 4.3 (0.3) | 4.3 (0.3) |  | 5.5  (26.0) | 5.6 (1.0) | 4.5  (0.8) |
|  | **Statistics** | | | | | | | | | | |
| R^2^ | 0.99 | 0.99 | 0.99 |  | 0.98 | 0.98 | 0.98 |  | 0.90 | 0.90 | 0.89 |
| RSQ | 0.010 | 0.009 | 0.010 |  | 0.0156 | 0.0132 | 0.0134 |  | 0.056 | 0.056 | 0.061 |
| # fit param | 6 | 4 | 3 |  | 6 | 4 | 3 |  | 6 | 4 | 3 |
| # data | 24 | 24 | 24 |  | 24 | 24 | 24 |  | 24 | 24 | 24 |
| BIC | -169 | -177 | -177 |  | -157 | -167 | -170 |  | -127 | -133 | -134 |

In all model versions (Table S1), pool sizes were constrained by the known pool size ratios: sucrose:fructan; sucrose:fructose; glucose:fructose (see Materials & Methods). Rate constant (*k*) estimates not significantly deviating from 0 are denoted by ns (not significant). RSQ, residual sum of squares; # fit param, number of fitting parameters; # data, number of data points; BIC, Bayesian information criterion. The results of the optimized rate constants and the corresponding fluxes are given in bold.

**Supporting Table S5‑1.** Pairwise comparisons of sucrose partitioning fractions (Table 3) within each CO₂ treatment.

| **Within CO₂ treatment** | **Comparison** | ***P*‑value**  **(z‑test, approximate)** |
| --- | --- | --- |
| 200 μmol mol^-1^ | growth and respiration vs fructan synthesis | 0.0258 |
|  | growth and respiration vs hydrolysis | 0.352 |
|  | fructan synthesis vs hydrolysis | 0.0058 |
| 400 μmol mol^-1^ | growth and respiration vs fructan synthesis | 0.230 |
|  | growth and respiration vs hydrolysis | 0.161 |
|  | fructan synthesis vs hydrolysis | 0.857 |
| 800 μmol mol^-1^ | growth and respiration vs fructan synthesis | 0.711 |
|  | growth and respiration vs hydrolysis | 0.826 |
|  | fructan synthesis vs hydrolysis | 0.944 |

*P*‑values were derived from two‑sample z‑tests assuming independence. Because the three fractions sum to 1, they are correlated; therefore, these within‑treatment comparisons are approximate and serve as an exploratory guide.

**Supporting Table S5‑2.** Pairwise *P*‑values from two‑sample z‑tests comparing each parameter in Table 3 among three CO_2_ concentrations (200, 400 and 800 μmol mol⁻¹).

| **Parameter** | **200 vs 400** | **200 vs 800** | **400 vs 800** |
| --- | --- | --- | --- |
| Sucrose half‑life | 0.724 | 0.823 | 0.655 |
| Fructan half‑life | 0.682 | 0.774 | 0.943 |
| Glucose half‑life | 0.346 | 0.170 | 0.493 |
| Fructose half‑life | 0.239 | 0.413 | 0.815 |
| F_in_ (= F_out_) | **0.0073** | **0.0073** | 0.146 |
| F_12_ (= F_24_) | **0.00035** | **0.0030** | 0.074 |
| F_13_ | 0.317 | **0.0143** | **0.0357** |
| F_14_ | 0.267 | 0.338 | 0.170 |
| F_31_ (= F_41_) | 0.424 | **0.0143** | **0.0291** |
| to growth and respiration | 0.607 | 0.609 | 0.297 |
| to fructan synthesis | **0.0455** | 0.171 | 0.761 |
| to hydrolysis | 0.137 | 0.517 | 0.813 |
| Residence time | 0.152 | **0.034** | 0.175 |

All *P*‑values were calculated using two‑sample z‑tests based on parameter estimates and their standard errors. CO₂ concentration was treated as a discrete factor with three levels. Bold values indicate significant differences at *P* < 0.05.

**Supporting Table S6** Results of a one-way ANOVA testing the effect of time on carbohydrate content per unit leaf area at each atmospheric CO_2_ concentration during dynamic labelling.

| **Atmospheric CO_2_ concentration** | **Parameter** | ***F*-value** | ***P*-value** |
| --- | --- | --- | --- |
| 200 μmol mol^-1^ |  |  |  |
|  | Fructan | 1.03 | 0.43 |
|  | Sucrose | 1.50 | 0.24 |
|  | Fructose | 1.46 | 0.25 |
|  | Glucose | 1.98 | 0.13 |
| 400 μmol mol^-1^ |  |  |  |
|  | Fructan | 0.16 | 0.98 |
|  | Sucrose | 0.84 | 0.53 |
|  | Fructose | 0.81 | 0.55 |
|  | Glucose | 0.77 | 0.58 |
| 800 μmol mol^-1^ |  |  |  |
|  | Fructan | 0.55 | 0.73 |
|  | Sucrose | 0.86 | 0.53 |
|  | Fructose | 1.61 | 0.21 |
|  | Glucose | 1.96 | 0.13 |

**Supporting Table S7** Shoot and root growth rates of plants during the final three weeks of the experiment and the fraction of WSC in the leaf growth and differentiation zones (LGDZ; Baca Cabrera et al. (2020), analyzed in the same experiment) relative to whole shoot biomass.

|  | **Atmospheric CO_2_ concentration (μmol mol^-1^)** | | |
| --- | --- | --- | --- |
|  | 200 | 400 | 800 |
| Growth rate |  |  |  |
| Shoot (g C plant^-1^ d^-1^) | 0.039^a^ (0.005) | 0.051^b^ (0.004) | 0.059^b^ (0.004) |
| Root (g C plant^-1^ d^-1^) | 0.002^a^ (0.002) | 0.002^a^ (0.002) | 0.003^a^ (0.003) |
| LGDZ WSC fraction | 0.091 (0.004) | 0.070 (0.004) | 0.070 (0.002) |

Growth rate was determined as the slope of the linear regression of carbon mass over time between days 49 and 63 for shoots and roots. Harvests were performed at the beginning and end of the dark period on these days. For each harvest, two random replicates of six plants each were collected from each growth chamber. For the methodology of LGDZ sampling and analysis, see Baca Cabrera et al. (2020).

**Supporting Table S8** Water-soluble carbohydrate concentrations (WSC: sucrose, fructan, glucose and fructose) in whole shoot biomass of perennial ryegrass (*Lolium perenne* L) as affected by atmospheric CO_2_ concentration during growth.

|  | Atmospheric CO_2_ concentration (μmol mol^-1^) | | |
| --- | --- | --- | --- |
|  | 200 | 400 | 800 |
| Sucrose (mg C g^-1^ biomass C) | 30.8^a^ (2.9) | 30.0^a^ (1.2) | 29.8^a^ (1.1) |
| Fructan (mg C g^-1^ biomass C) | 350.6^a^ (16.5) | 451.2^b^ (9.9) | 503.9^c^ (26.4) |
| Glucose (mg C g^-1^ biomass C) | 17.9^a^ (1.9) | 14.5^b^ (0.6) | 14.6^b^ (0.5) |
| Fructose (mg C g^-1^ biomass C) | 18.5^a^ (1.3) | 19.0^a^ (0.8) | 22.9^b^ (1.1) |

Means ± SE of 44-48 replicates sampled during the labelling experiment. Different superscript letters within a row indicate statistically significant differences among CO_2_ treatments at *P* <0.001.
